# Harnessing the Therapeutic Potential of Stem Cells in the Management of Chronic Obstructive Pulmonary Disease: A Comprehensive Review

**DOI:** 10.7759/cureus.44498

**Published:** 2023-08-31

**Authors:** Parth V Singh, Prateesh V Singh, Ashish Anjankar

**Affiliations:** 1 Internal Medicine, Indira Gandhi Government Medical College, Nagpur, IND; 2 Medicine and Surgery, Jawaharlal Nehru Medical College, Datta Meghe Institute of Higher Education and Research, Wardha, IND; 3 Biochemistry, Jawaharlal Nehru Medical College, Datta Meghe Institute of Higher Education and Research, Wardha, IND

**Keywords:** therapeutic potential challenges, tissue regeneration, immunomodulation, lung repair, regenerative medicine, stem cells, copd

## Abstract

Chronic obstructive pulmonary disease (COPD) is a prevalent and debilitating respiratory condition with limited treatment options. Stem cell therapy has emerged as a promising approach for COPD management due to its regenerative and immunomodulatory properties. This review article aims to comprehensively explore the therapeutic potential of stem cells in COPD management. The introduction provides background on COPD, highlighting its impact on health and the need for novel therapies. The different types of stem cells relevant to COPD, including embryonic stem cells, adult stem cells, and induced pluripotent stem cells, are described along with their properties and characteristics. The pathogenesis of COPD is discussed, emphasizing the key mechanisms involved in disease development and progression. Subsequently, the role of stem cells in tissue repair, regeneration, and immunomodulation is examined, highlighting their ability to address specific pathological processes in COPD. Mechanisms of action, such as paracrine signaling, immunomodulation, anti-inflammatory effects, and tissue regeneration, are explored. The interaction between stem cells and the host environment, which promotes lung repair, is also discussed. Challenges in stem cell therapy for COPD, including optimal cell sources, delivery methods, safety, and efficacy, are identified. Regulatory considerations and the importance of standardization are emphasized. Potential strategies for optimizing the therapeutic potential of stem cells in COPD management, such as combination therapies and preconditioning techniques, are outlined. Emerging trends and future directions are highlighted, including advanced cell engineering and patient-specific induced pluripotent stem cells. In conclusion, stem cell therapy holds significant promise for COPD management, addressing the limitations of current treatments. Continued research and development are necessary to overcome challenges, optimize therapies, and realize stem cells' full potential in improving the lives of patients with COPD.

## Introduction and background

Chronic obstructive pulmonary disease (COPD) is a progressive respiratory disorder characterized by persistent airflow limitation and respiratory symptoms such as coughing, wheezing, and shortness of breath. It encompasses a group of lung conditions, primarily chronic bronchitis and emphysema, often caused by long-term exposure to harmful substances such as cigarette smoke or occupational pollutants. Chronic obstructive pulmonary disease is a major global health issue, affecting millions of people worldwide and resulting in significant morbidity and mortality [1-3].

Despite advancements in the management of COPD, the disease remains a significant burden on individuals and healthcare systems. Current treatment strategies aim to alleviate symptoms, reduce exacerbations, and improve quality of life. However, there are several challenges to effectively managing COPD. These include the limited reversibility of lung damage, the progressive nature of the disease, and the incomplete response to existing therapies in some patients. As a result, there is a critical need for novel therapeutic approaches that can modify the underlying disease processes and provide long-term benefits [4].

Stem cells are undifferentiated cells with the unique ability to self-renew and differentiate into specialized cell types. They hold tremendous promise in regenerative medicine due to their potential to repair and regenerate damaged tissues. Stem cells can be classified into different types, including embryonic stem cells, adult stem cells, and induced pluripotent stem cells, each with its distinct properties and characteristics. Stem cells have shown great potential in various fields of medicine, including cardiovascular diseases, neurological disorders, and tissue repair [5].

This review article aims to comprehensively explore stem cells' therapeutic potential in managing COPD. By examining the current understanding of COPD pathogenesis and the mechanisms of stem cells, we aim to elucidate how stem cell therapy can address the underlying disease processes and potentially provide novel treatment options for COPD. Additionally, we discuss the challenges and future perspectives of harnessing the therapeutic potential of stem cells for COPD management. Through this review, we hope to contribute to the growing body of knowledge in the field and stimulate further research in this promising area of regenerative medicine.

## Review

Types of stem cells relevant to COPD

Various types of stem cells have been investigated for their potential in COPD management. These include embryonic stem cells (ESCs), adult stem cells, and induced pluripotent stem cells (iPSCs) [6].

Embryonic Stem Cells

Derived from early-stage embryos, ESCs can differentiate into any cell type in the body. They are characterized by their pluripotency, meaning they can give rise to cells from all three germ layers. Embryonic stem cells have attracted significant attention due to their unlimited proliferative capacity and broad differentiation potential [7].

*Adult Stem Cells* 

Also known as tissue-specific or somatic stem cells, adult stem cells are present in various tissues and organs throughout the body, including the lungs. These cells play a vital role in tissue maintenance and repair. Adult stem cells relevant to COPD include mesenchymal stem cells (MSCs) derived from bone marrow, adipose tissue, or other sources and epithelial stem cells residing in the airways [8].

Induced Pluripotent Stem Cells

These are generated by reprogramming adult somatic cells, such as skin cells, to a pluripotent state. This reprogramming is achieved by introducing specific transcription factors into the cells, allowing them to regain pluripotency. Induced pluripotent stem cells have characteristics similar to ESCs, including self-renewal and the ability to differentiate into various cell types [9].

Properties and characteristics of these three types of stem cells

Embryonic Stem Cells

Embryonic stem cells derived from early-stage embryos are characterized by their pluripotency, i.e., the ability to differentiate into cells of all three germ layers: endoderm, mesoderm, and ectoderm. This remarkable ability to differentiate into various cell types makes ESCs highly valuable in regenerative medicine and tissue engineering [10].

Another distinctive feature of ESCs is their unlimited self-renewal capacity. These cells can proliferate indefinitely in culture, providing a potentially limitless source of cells for therapeutic applications. This ability to continuously divide and generate large quantities of ESCs is essential for scalability and the production of sufficient cells for transplantation or research purposes [11].

However, it is important to acknowledge the ethical considerations surrounding the use of ESCs. The extraction of ESCs involves the destruction of early-stage embryos, raising ethical concerns for some individuals and communities. Some see the destruction of embryos as ethically problematic due to their potential for human life. These ethical considerations have led to various regulations and guidelines governing the use of ESCs in research and therapy [12]. To address these ethical concerns and overcome the limitations associated with ESCs, researchers have explored alternative approaches, such as adult stem cells or iPSCs. Adult stem cells can be obtained from various adult tissues, and iPSCs can be generated by reprogramming adult cells to an embryonic-like state, offering potential alternatives that avoid the ethical concerns associated with ESCs.

Adult Stem Cells

Adult stem cells are a subset of stem cells found in specific tissues throughout the body and play a crucial role in tissue homeostasis and repair. Unlike ESCs, which can differentiate into any cell type in the body, adult stem cells have a more limited differentiation potential. They are typically multipotent or unipotent, meaning they can differentiate into a restricted range of cell types within their tissue of origin [13].

The tissue-specific nature of adult stem cells means they reside in specific organs or tissues, such as the bone marrow, adipose tissue, or the lungs. These stem cells are responsible for maintaining the health and function of their respective tissues by replenishing damaged or dying cells through their ability to differentiate into specialized cell types [14].

While adult stem cells may have a more restricted differentiation potential than ESCs, they can still differentiate into various cell types within their tissue of origin. For example, bone marrow-derived adult stem cells can produce various blood cell types, including red blood cells, white blood cells, and platelets. Similarly, adult stem cells found in adipose tissue can differentiate into adipocytes, which comprise fat tissue [15].

One advantage of adult stem cells is that they are relatively accessible compared to other types of stem cells. They can often be obtained from easily accessible bodily sources, such as bone marrow through a minimally invasive procedure or adipose tissue through liposuction. This accessibility makes adult stem cells a promising option for autologous transplantation, where a patient's stem cells are used for therapeutic purposes, reducing the risk of immune rejection [16].

Induced Pluripotent Stem Cells (iPSCs)

Induced pluripotent stem cells are a type of stem cell that holds great promise in regenerative medicine. They are generated by reprogramming adult somatic cells, such as skin cells, to a pluripotent state. They share similarities with ESCs in terms of their pluripotency, meaning they have the potential to differentiate into various cell types of the body [17].

One notable advantage of iPSCs is their patient-specific nature. They can be derived from a patient's cells, such as skin cells, and reprogrammed into iPSCs. This patient-specific approach offers potential advantages in minimizing immune rejection when iPSC-derived cells are used for transplantation or therapeutic purposes. Using a patient's cells reduces the risk of immune rejection, increasing the feasibility and safety of iPSC-based therapies [18].

However, it is important to acknowledge the challenges associated with iPSCs. Reprogramming adult somatic cells into iPSCs can be complex and inefficient. It requires introducing specific transcription factors that induce the cells to regain pluripotency. The reprogramming efficiency can vary; not all cells may successfully transition into iPSCs. Moreover, during the reprogramming process, genetic and epigenetic abnormalities are at risk. These abnormalities can affect the stability and functionality of iPSCs and pose potential risks in therapeutic applications [19].

Despite these challenges, ongoing research is focused on optimizing iPSC generation techniques and improving their safety and efficiency. Strategies such as non-integrating reprogramming methods, precise gene editing techniques, and rigorous quality control measures aim to enhance the reliability and clinical applicability of iPSCs [20].

Potential advantages and limitations of ESCs, adult stem cells, and iPSCs in COPD management

Advantages of Embryonic Stem Cells 

Robust differentiation potential: Embryonic stem cells can differentiate into various cell types of all three germ layers. This pluripotency makes them well-suited for generating diverse lung cell types, including alveolar, bronchial, and endothelial cells. By differentiating into these specific cell types, ESCs can potentially contribute to regenerating damaged lung tissue [21].

Extensive expansion in culture: Embryonic stem cells have a high proliferation capacity and can be expanded extensively. This characteristic is advantageous for generating many cells for transplantation or experimental purposes. Expanding ESCs in vitro allows for scalability and the production of sufficient lung-specific cells for potential therapeutic applications [15].

Limitations of Embryonic Stem Cells 

Ethical concerns: The use of ESCs raises ethical concerns due to their derivation from early-stage embryos. The extraction of ESCs typically involves the destruction of embryos, which is a point of ethical debate. These ethical considerations have led to certain legal and regulatory restrictions on using ESCs in some countries [22].

Risk of teratoma formation: One of the major limitations associated with ESCs is the potential risk of teratoma formation upon transplantation. Teratomas are tumors consisting of cells derived from all three germ layers that may develop from undifferentiated ESCs. This risk emphasizes the importance of rigorous quality control measures and safety assessments to minimize the potential for teratoma formation before considering clinical applications of ESCs [23].

Advantages of Adult Stem Cells

Adult stem cells offer several advantages in the context of COPD management. One major advantage is their relative accessibility from different tissue sources, such as bone marrow or adipose tissue, making them potentially suitable for autologous transplantation. This means that a patient's stem cells can be obtained and used for therapeutic purposes, minimizing the risk of immune rejection. Adult stem cells also possess immunomodulatory properties, allowing them to interact with the immune system and modulate its activity. They can secrete various factors, including cytokines and growth factors, which aid in tissue repair processes and promote the regeneration of damaged lung tissue. By modulating the immune response, adult stem cells can help create a favorable environment for tissue healing and mitigate excessive inflammation [24].

Limitations of Adult Stem Cells

Despite their advantages, adult stem cells have certain limitations that must be considered. One limitation is their limited differentiation potential compared to embryonic stem cells. Adult stem cells are often multipotent or unipotent, meaning they can differentiate into a limited range of cell types. This restricted differentiation potential may limit their ability to generate specific lung cell types required for complete tissue regeneration. Adult stem cells may also exhibit reduced proliferation and regenerative capacity in aged or diseased individuals. Factors such as age-related decline or comorbidities can negatively impact the function of adult stem cells and their regenerative potential. It is important to consider these limitations when evaluating the suitability of adult stem cells for therapeutic applications in COPD management [25].

Advantages of iPSCs

Induced pluripotent stem cells offer several advantages in regenerative medicine and disease research. One significant advantage is their potential to provide a personalized approach to treatment. They can be derived from a patient's cells, such as skin cells, which reduces the risk of immune rejection upon transplantation. This personalized aspect allows the development of patient-specific therapies tailored to individual characteristics and needs [26].

Another advantage of iPSCs is their ability to differentiate into various cell types. Through controlled differentiation protocols, iPSCs can be guided to develop into specific cell lineages, including those relevant to the affected tissues or organs in a particular disease. This differentiation potential holds promise for disease modeling, as iPSCs can generate disease-specific cell lines that mimic the characteristics and behavior of the patient's cells. It also provides a valuable platform for drug discovery and testing, enabling researchers to study the effects of potential therapies on patient-derived cells before moving to clinical trials [27].

Limitations of iPSCs

While iPSCs offer exciting possibilities, several limitations need to be addressed. One of the key challenges is reprogramming efficiency. Reprogramming adult somatic cells into iPSCs can be relatively inefficient, with low success rates. This inefficiency hinders the generation of sufficient iPSCs for therapeutic purposes or research studies. Enhancing the efficiency of reprogramming techniques is an ongoing area of investigation [28].

Another important consideration is the risk of genetic and epigenetic abnormalities during reprogramming. The reprogramming process involves introducing specific transcription factors to reset the adult cells back to a pluripotent state. However, this reprogramming can induce genetic mutations or epigenetic changes that alter the behavior or function of iPSCs. These abnormalities may impact the safety and efficacy of iPSC-based therapies and need to be carefully monitored and controlled [29].

Pathogenesis of COPD and the rationale for stem cell therapy

Key Mechanisms Involved in COPD Development and Progression

The pathogenesis of COPD involves multiple interconnected mechanisms that contribute to the development and progression of the disease. These mechanisms include chronic inflammation, oxidative stress, tissue destruction, impaired tissue repair, and airway remodeling. Prolonged exposure to noxious particles, such as cigarette smoke, triggers an inflammatory response in the airways, leading to the recruitment of immune cells and the release of pro-inflammatory mediators. This chronic inflammation, along with the generation of reactive oxygen species, damages the lung tissue, impairs lung function, and contributes to the characteristic airflow limitation observed in COPD [30].

Role of Stem Cells in Tissue Repair, Regeneration, and Immunomodulation

Stem cells possess unique properties that make them attractive candidates for therapeutic interventions in COPD. They can self-renew and differentiate into various cell types, including lung-specific cells. Stem cells can contribute to tissue repair and regeneration by replacing damaged cells and promoting healing. They also exert immunomodulatory effects by regulating the immune response, reducing inflammation, and promoting tissue homeostasis [31].

Mesenchymal stem cells are particularly interesting due to their immunomodulatory properties. They interact with immune cells and modulate their activity, promoting an anti-inflammatory environment. They can secrete various factors, including anti-inflammatory cytokines, growth factors, and extracellular vesicles, inhibiting pro-inflammatory responses and promoting tissue repair processes [32].

Epithelial stem cells in the airways play a crucial role in maintaining the integrity of the respiratory epithelium. These cells have the potential to differentiate into various lung epithelial cell types and contribute to the repair of damaged airway tissue. Stem cells can also promote angiogenesis, forming new blood vessels, which is important for delivering oxygen and nutrients to the injured lung tissue [33].

How Stem Cell Therapy Can Address Specific Pathological Processes in COPD

Stem cell therapy holds promise for addressing specific pathological processes in COPD by targeting multiple aspects of the disease's pathogenesis. One key mechanism is tissue repair and regeneration, where stem cells can differentiate into lung-specific cells and replace damaged or lost cells. This contributes to the restoration of lung function, improves the structural integrity of the airways, and reduces airflow limitation [34].

Another important mechanism is immunomodulation, particularly mediated by MSCs. Stem cells can modulate the immune response by inhibiting excessive inflammation and promoting an anti-inflammatory environment. This immunomodulatory effect helps alleviate the chronic inflammation associated with COPD, reducing lung tissue damage and promoting a more favorable environment for repair [35].

Stem cells also exhibit anti-fibrotic effects, which are crucial in COPD management as fibrosis is a characteristic feature of the disease. Stem cells can inhibit the activation of fibroblasts and myofibroblasts, thereby reducing excessive collagen deposition and preventing the progression of fibrotic remodeling in the lungs [36]. Additionally, stem cells can promote angiogenesis, and the formation of new blood vessels. This enhanced vascularization improves blood supply to the damaged lung tissue, enhancing oxygenation and supporting tissue repair processes. By harnessing these mechanisms, stem cell therapy offers a potential strategy to modify the course of COPD, mitigate disease progression, and improve patients' lung function and quality of life. However, further research is needed to optimize stem cell delivery methods, determine the ideal cell type and dosage, and address safety concerns before widespread clinical implementation. Continued research efforts in this field will provide valuable insights and pave the way for the successful clinical translation of stem cell therapies in COPD management [37].

Mechanisms of stem cells in COPD management

An examination of the different mechanisms by which stem cells exert their therapeutic effects in COPD revealed a multifaceted approach that promotes lung repair and mitigates the pathological processes associated with the disease [38].

First, stem cells exert their effects through paracrine signaling, which involves the secretion of bioactive factors and signaling molecules. These include cytokines, growth factors, chemokines, and extracellular vesicles, which act on neighboring cells in the lung tissue. Paracrine signaling promotes tissue repair and regeneration by influencing various cell types, such as immune, epithelial, and endothelial cells. The signals released by stem cells stimulate the activation of endogenous repair mechanisms, promote cell proliferation and migration, and modulate the inflammatory response [39].

Immunomodulation is another important mechanism through which stem cells exert their therapeutic effects in COPD. Particularly, MSCs have immunomodulatory properties that enable them to interact with immune cells and regulate their activity. Mesenchymal stem cells can inhibit the proliferation and activation of immune cells, including T cells and macrophages, and induce the generation of regulatory T cells. This immunomodulatory effect helps to reduce the chronic inflammation present in COPD, alleviate tissue damage, and create a more favorable environment for tissue repair and regeneration [40].

Furthermore, stem cells possess anti-inflammatory effects that contribute to the therapeutic benefits in COPD. Stem cells can secrete anti-inflammatory cytokines and factors, such as interleukin-10 (IL-10), transforming growth factor-beta (TGF-β), and prostaglandin E2 (PGE2). These molecules counteract pro-inflammatory signaling pathways, inhibit immune cell recruitment and activation, and suppress inflammatory mediators' production. By reducing inflammation, stem cells help minimize tissue damage and create an environment conducive to healing and repair [41].

In addition to their paracrine signaling, immunomodulation, and anti-inflammatory effects, stem cells play a crucial role in tissue regeneration. Stem cells can differentiate into various cell types, including lung-specific cells such as epithelial cells, endothelial cells, and MSCs. When delivered to damaged lung tissue, stem cells can replace lost or damaged cells, contribute to the regeneration of lung structures, and promote functional recovery. Additionally, stem cells can stimulate endogenous tissue-resident stem cells, enhancing their activity and regenerative potential [42].

Paracrine Signaling, Immunomodulation, Anti-inflammatory Effects, and Tissue Regeneration

Paracrine signaling: Stem cells release various bioactive factors and signaling molecules, such as cytokines, growth factors, chemokines, and extracellular vesicles. These paracrine factors can influence neighboring cells, including immune cells, epithelial cells, and endothelial cells. Paracrine signaling promotes tissue repair, modulates the immune response, reduces inflammation, and stimulates endogenous regenerative processes [43].

Immunomodulation: Stem cells, particularly MSCs, possess immunomodulatory properties. They can interact with immune cells, such as T cells, B cells, macrophages, and dendritic cells, and modulate their activity. Mesenchymal stem cells can suppress the proliferation and activation of immune cells, shift the immune response from pro-inflammatory to anti-inflammatory, and promote the generation of regulatory T cells. This immunomodulatory effect helps to dampen chronic inflammation, reduce tissue damage, and create a more favorable environment for tissue repair and regeneration [44].

Anti-inflammatory effects: Stem cells can directly secrete anti-inflammatory cytokines and factors, such as IL-10, TGF-β, and PGE2. These anti-inflammatory molecules help counteract the chronic inflammation observed in COPD by inhibiting pro-inflammatory signaling pathways, reducing the recruitment and activation of immune cells, and suppressing the production of inflammatory mediators [45].

Tissue regeneration: Stem cells can differentiate into lung-specific cell types, including epithelial cells, endothelial cells, and MSCs. When delivered to damaged lung tissue, stem cells can replace lost or damaged cells, contribute to the regeneration of lung structures, and promote functional recovery. Additionally, stem cells can stimulate endogenous tissue-resident stem cells, enhancing their activity and regenerative potential [46].

Current Understanding of How Stem Cells Interact With the Host Environment to Promote Lung Repair

Stem cells interact with the host environment in several ways to promote lung repair. After transplantation or activation in situ, stem cells return to the injured lung tissue, guided by chemotactic factors and adhesion molecules. Once in the damaged area, stem cells interact with local cells and the extracellular matrix through cell-cell contacts and the secretion of paracrine factors [47].

Stem cells integrate into the microenvironment and can influence neighboring cells through paracrine signaling. They secrete various factors that regulate cell proliferation, differentiation, migration, and survival. These factors can promote resident lung stem cell activation, modulate immune cell behavior, and stimulate angiogenesis and tissue remodeling processes [48].

Furthermore, stem cells can directly interact with immune cells, modulating their activity and function. This interaction occurs through cell-cell contact and the release of soluble factors, suppressing pro-inflammatory responses and inducing anti-inflammatory and tissue repair-promoting processes [49].

The specific mechanisms underlying stem cell-host interactions in the context of COPD are still being elucidated, and further research is needed to fully understand the dynamic interplay between stem cells and the host environment during lung repair processes. Improved knowledge in this area will contribute to optimizing stem cell-based therapies for COPD management [50].

Challenges and future perspectives

Identification of Key Challenges and Obstacles in Stem Cell Therapy for COPD

Stem cell therapy for COPD is a promising approach, but it faces several challenges that must be addressed for successful implementation. First, identifying the optimal cell source is crucial. Different types of stem cells, such as ESCs, adult stem cells, and iPSCs, have varying therapeutic potential. Determining the most suitable and effective cell source for COPD treatment is an ongoing area of research [51].

Second, developing effective delivery methods is a challenge. Delivering stem cells to damaged lung tissue in a targeted and efficient manner is essential for optimal therapeutic outcomes. Factors like cell survival, engraftment, and homing must be carefully considered to enhance the efficacy of stem cell therapies in COPD [52].

Ensuring the safety and long-term efficacy of stem cell therapies is another critical concern. Understanding and mitigating the potential risks associated with stem cell therapy is essential. One such risk is tumorigenicity, where stem cells may undergo uncontrolled growth and form tumors. Immunogenicity, or the potential for an immune response against the transplanted stem cells, is also a consideration. Developing strategies to minimize these risks and ensure stem cell therapy's long-term safety and efficacy is of utmost importance [31].

Regulatory Considerations and Standardization of Stem Cell Therapies

Regulatory considerations and standardization play a vital role in the successful clinical translation of stem cell therapies, including those for COPD. Several key aspects are involved in ensuring the safety, efficacy, and ethical implementation of these therapies.

Regulatory approval: Like any other therapeutic intervention, stem cell therapies for COPD must undergo rigorous regulatory evaluation and approval processes. This ensures that the therapies meet quality, safety, and efficacy standards. Compliance with regulatory guidelines and demonstrating well-designed clinical trials are crucial steps for successfully translating stem cell therapies into clinical practice [53].

Quality control and manufacturing: Standardizing stem cell production and manufacturing processes is essential to ensuring consistent and reliable cell therapies. Developing robust quality control measures, including thorough characterization and potency assays, is necessary for maintaining product quality, safety, and reproducibility. Establishing standardized protocols and procedures for stem cell production, handling, and storage can minimize variations between batches and enhance the reliability of the therapies [54].

Ethical considerations: Stem cell therapies, particularly those utilizing embryonic stem cells, raise ethical concerns that must be carefully addressed. Adhering to established ethical guidelines and regulations is essential throughout the development and application of stem cell therapies. Ensuring proper informed consent from patients and ethical oversight of stem cell research are crucial aspects that uphold ethical standards in this field [55].

Potential Strategies for Optimizing the Therapeutic Potential of Stem Cells in COPD Management

Combination therapies: Combining stem cell therapy with other treatment modalities, such as pharmacological interventions or gene therapy, may have synergistic effects and enhance therapeutic outcomes. By targeting multiple aspects of COPD pathogenesis simultaneously, combination therapies have the potential to provide more comprehensive and effective treatment. Understanding the potential interactions between different therapeutic approaches and designing appropriate combination strategies is an ongoing investigation [56].

Preconditioning techniques: Preconditioning stem cells before transplantation can enhance their therapeutic properties. This can be achieved through various techniques such as genetic modification, exposure to specific growth factors, or manipulating oxygen levels. Preconditioning strategies aim to improve stem cell survival, engraftment, and their ability to modulate the host environment. By priming the cells before transplantation, their therapeutic potential can be optimized, leading to improved outcomes [57].

Biomaterials and scaffolds: Incorporating stem cells into biomaterials or scaffolds can provide structural support, enhance cell retention, and create a favorable microenvironment for cell survival and differentiation. Biomaterials and scaffolds can mimic the architecture and properties of lung tissue, providing a suitable niche for stem cell growth and function. This approach can improve the delivery and localization of stem cells to the targeted lung regions, enhancing their therapeutic effects [58].

Developing suitable biomaterials and scaffolds that closely resemble the lung tissue architecture is crucial for optimizing stem cell therapies in COPD. Advances in biomaterial and tissue engineering techniques enable the creation of biomimetic platforms that can better support stem cell survival, differentiation, and integration within damaged lung tissue [59].

Exploring these strategies, namely combination therapies, preconditioning techniques, and biomaterials and scaffolds, can significantly enhance the therapeutic potential of stem cells in COPD management. Further research and development efforts in these areas will contribute to refining and optimizing stem cell-based therapies, leading to improved outcomes and a greater impact on COPD patients' lives [60].

Emerging Trends and Future Directions in the Field

Advanced cell engineering: Genetic modification and engineering techniques are being investigated to enhance the therapeutic potential of stem cells in COPD. This includes gene editing or manipulating stem cell properties to target specific disease mechanisms. By modifying stem cells, researchers aim to optimize their regenerative capabilities and tailor them to address the specific needs of COPD patients [61].

Patient-specific iPSCs: Induced pluripotent stem cells generated from a patient's cells offer the possibility of personalized therapies for COPD. These iPSCs can be reprogrammed to differentiate into lung-specific cells, providing a tailored approach to treatment. Utilizing patient-specific iPSCs can minimize the risk of immune rejection, as the cells are derived from the patient's body [62].

Precision medicine approaches: Chronic obstructive pulmonary disease is a heterogeneous disease with varying characteristics and subtypes. Advancements in understanding the heterogeneity of COPD enable researchers to identify specific patient subgroups. This knowledge can inform the development of targeted stem cell therapies tailored to individual patient characteristics. Precision medicine approaches allow for personalized treatment strategies, improving treatment outcomes and patient stratification [63].

Continued research, clinical trials, and interdisciplinary collaborations are vital to address the challenges associated with stem cell therapy for COPD and optimize its effectiveness. These efforts will contribute to developing safe and effective stem cell-based treatments for COPD, ultimately leading to improved patient outcomes and enhanced quality of life. The future of stem cell therapy in COPD management holds great promise, and ongoing exploration of emerging trends and advancements will shape the future of this field [64].

## Conclusions

The therapeutic potential of stem cells for managing COPD offers a promising avenue to address the challenges associated with this debilitating condition. Stem cells possess unique properties, including their ability to repair damaged tissue, modulate the immune response, and promote regeneration, making them attractive candidates for regenerative medicine in COPD. The mechanisms of action, such as paracrine signaling, immunomodulation, anti-inflammatory effects, and tissue regeneration, highlight the diverse ways stem cells can address the complex pathological processes involved in COPD. Although challenges to overcome include optimizing cell sources, refining delivery methods, ensuring safety and long-term efficacy, and addressing regulatory considerations, continued research and development in this field are crucial. Advancements in stem cell therapy will deepen our understanding of stem cell-host interactions, facilitate personalized approaches, and contribute to advancing precision medicine in COPD management. By harnessing the therapeutic potential of stem cells, we can envision a future where COPD patients experience improved outcomes, enhanced quality of life, and greater hope for managing this chronic condition.
